# Functional *MPO* Polymorphisms and Haplotypes Affect Both Myeloperoxidase Levels and Association with Hypertensive Disorders of Pregnancy

**DOI:** 10.3390/ijms26157071

**Published:** 2025-07-23

**Authors:** Daniela Alves Pereira, Marcelo Rizzatti Luizon, Ricardo Carvalho Cavalli, Jose Eduardo Tanus-Santos, Valéria Cristina Sandrim

**Affiliations:** 1 Department of Physiology and Pharmacology, Institute of Biosciences of Botucatu, Universidade Estadual Paulista (UNESP), Distrito Rubiao Junior, Botucatu 18680-000, SP, Brazil; da.pereira@unesp.br (D.A.P.); mrluizon@icb.ufmg.br (M.R.L.); 2 Department of Genetics, Ecology and Evolution, Institute of Biological Sciences, Federal University of Minas Gerais, Belo Horizonte 31270-901, MG, Brazil; 3 Department of Gynecology and Obstetrics, Faculty of Medicine of Ribeirao Preto, University of Sao Paulo, Av. Bandeirantes, 3900, Ribeirao Preto 14049-900, SP, Brazil; rcavalli@fmrp.usp.br; 4 Department of Pharmacology, Faculty of Medicine of Ribeirao Preto, University of Sao Paulo, Av. Bandeirantes, 3900, Ribeirao Preto 14049-900, SP, Brazil; tanus@fmrp.usp.br

**Keywords:** preeclampsia, myeloperoxidase, MPO, genetic polymorphisms

## Abstract

Preeclampsia (PE) shares common pathophysiological mechanisms with cardiovascular diseases, including endothelial dysfunction and exacerbated inflammatory response. Myeloperoxidase (MPO) has been suggested as a biomarker for cardiovascular risk, and its circulating levels are contradictory in PE. Elevated levels of MPO can promote host tissue damage and trigger vascular injury. *MPO* gene polymorphisms affect circulating MPO levels under different conditions. To date, no studies have investigated whether MPO polymorphisms influence MPO levels in hypertensive disorders of pregnancy. In this study, we examined the impact of two specific *MPO* polymorphisms—rs2243828 and rs2071409—and their associated haplotypes on MPO levels. We also explored their potential association with gestational hypertension (GH) and preeclampsia (PE). Our study included 136 healthy pregnant women (HP), including 118 with GH and 140 with PE. Genotyping was performed using TaqMan allele discrimination assays, and MPO levels were quantified using an ELISA assay. The TT genotype of the rs2243828 polymorphism was associated with lower MPO concentration in GH, and the CC genotype presented a higher frequency in the GH group than the HP group. The AC+CC rs2071409 polymorphism was associated with lower MPO concentration in GH. We also found that the ‘C, C’ haplotype was less frequent and was associated with lower MPO concentration in PE. Our findings suggest that both rs2243828 and rs2071409 polymorphisms might contribute to MPO levels in GH and that the haplotype ‘C, C’ formed by them may protect against PE.

## 1. Introduction

Hypertensive disorders of pregnancy (HDP) are among the primary causes of maternal and fetal complications and death [1]. Gestational hypertension (GH) and preeclampsia (PE) are the most frequent types, both marked by the onset of high blood pressure at or after 20 weeks of gestation. What differentiates PE from GH is the presence of proteinuria or signs of organ dysfunction, such as abnormal liver function or neurological symptoms [2,3]. Despite several studies, the cause of these disorders remains unclear, and there are no available treatments that reverse the pathophysiological alterations; however, antihypertensive treatment can be used to decrease complications associated with high blood pressure [4,5].

Found in large amounts in neutrophils, myeloperoxidase (MPO) is a key component of the innate immune system, providing a defense against invading pathogens. MPO is responsible for catalyzing the formation of highly reactive haloacid substances formed from halogens in the presence of hydrogen peroxide [6]. However, these reactive compounds can also promote host tissue damage and the development of disease [7,8]. This enzyme can trigger vascular injury, mostly because of its oxidative and proinflammatory properties, and has been linked to several cardiovascular diseases (CVDs) [9,10]. Studies have confirmed the presence of neutrophils and MPO in the decidua basalis of the placenta [11]. Additionally, increased *MPO* expression on the surface of circulating neutrophils has been reported during pregnancy [11]; however, its exact levels and function during implantation and placentation are still not well understood. In this context, myeloperoxidase is associated with impaired bioavailability of nitric oxide (NO) [10,12], a molecule produced by endothelial cells and clinically reduced in PE patients [13,14].

Preeclampsia (PE) has been linked to a greater risk of developing CVDs later in life, suggesting that PE and CVD may share underlying pathophysiological pathways [15,16]. In support of this, epidemiological research has demonstrated that elevated MPO levels are associated with increased CVD risk, as reviewed in previous studies [17]. In our earlier work, we observed that MPO levels were elevated in patients with PE and GH who had not received antihypertensive treatment [9]. However, the literature on MPO levels in PE remains inconsistent, with some studies reporting increased levels and others showing no significant difference between groups [18,19,20,21,22,23,24].

The *MPO* gene is known to be polymorphic, and single-nucleotide polymorphisms (SNPs) are common genetic variations that can influence gene function [25]. In this regard, only a limited number of studies have explored the relationship between *MPO* polymorphisms and the risk of developing PE [26,27]. To date, no prior research has investigated whether these polymorphisms or their haplotypes impact plasma MPO levels in hypertensive pregnancy disorders. It is plausible that individuals with gestational hypertension (GH) or PE who carry certain *MPO* genotypes or haplotypes may exhibit differing levels of plasma MPO.

In this context, *MPO* SNPs c.–765T>C (rs2243828) and c.–765T>C (rs2243828) are likely to affect the transcription factor binding site according to Score 1f at RegulomeDB [28], and it is also classified as a candidate cis-Regulatory Element (cCRE) according to the Encyclopedia of DNA Elements (ENCODE) data [29].

In this study, we investigated whether these *MPO* SNPs, rs2071409 and rs2243828, along with the haplotypes formed by their allele combinations, influence plasma levels of myeloperoxidase in healthy pregnant (HP) women and in those with GH and PE, and whether they are linked to susceptibility to GH and PE.

## 2. Results

The clinical characteristics of the patients enrolled for the genotype analysis are shown in Table 1. Women with HP, GH, and PE had comparable ethnicities (percentage white), rates of current smoking, hemoglobin levels, and red blood cell counts (*p* > 0.05). Both the PE and GH groups exhibited higher systolic and diastolic blood pressures and higher body mass index (BMIs) than the HP group, with PE patients presenting higher diastolic blood pressure than GH patients, while GH patients presented higher BMIs than the PE group (both *p* < 0.05). It is important to note that most patients were taking antihypertensive medication. The proportions of GH and PE patients receiving such treatment are detailed in Table 1, with nifedipine being taken more frequently by PE patients when compared to GH patients. PE and GH patients were older than HP patients (*p* < 0.05). We found lower neutrophils in GH and PE groups than in HP subjects, as well as lower gestational age at delivery (GAD) in GH and PE groups when compared to HP patients, and lower GAD in PE than in GH patients (all *p* < 0.05). Gestational age at sampling (GAS) was lower in PE than in GH and HP groups. Regarding plasma nitrite and whole blood nitrite, GH and PE presented lower plasma nitrite when compared to HP patients, and PE patients presented lower whole blood nitrite than the other two groups (all *p* < 0.05). PE patients presented lower newborn weights and more significant proteinuria (all *p* < 0.05).

Table 2 presents the distribution of genotypes and alleles. For each polymorphism, genotype frequencies were consistent with Hardy–Weinberg equilibrium. The CC genotype for the c.–765T>C (rs2243828) polymorphism was found at a higher frequency in the GH group than in the HP group (*p* < 0.05). Conversely, there were no statistically significant differences in the genotype or allele frequencies of the g.9890A>C (rs2071409) polymorphism among the study groups (*p* > 0.05). The distribution of haplotypes is shown in Table 3. The ‘C, C’ haplotype appeared significantly more frequently in the HP group than in the PE group (*p* = 0.025). In contrast, no significant difference was observed between the HP and GH groups (*p* > 0.05).

We examined the effects of *MPO* genotypes (Table 4) and haplotypes (Table 5) on plasma MPO levels. We found that GH patients carrying the TT genotype for the c.–765T>C (rs2243828) polymorphism and the AC+CC genotypes for the g.9890A>C (rs2071409) polymorphism showed lower levels of MPO than patients carrying the TC+CC genotypes (*p* < 0.0001) and the AA genotype (*p* = 0.02), respectively.

**Figure 1:**
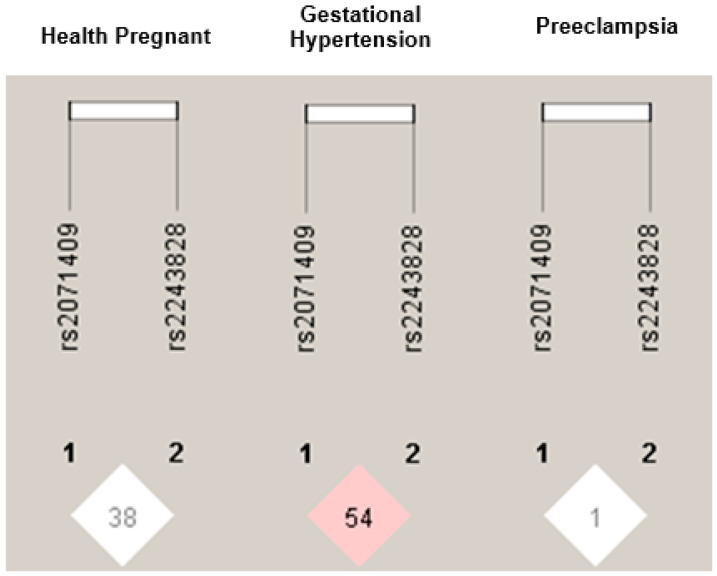
LD plots for the *MPO* gene SNPs (rs2071409 and rs2243828) are shown, identified by their dbSNP IDs. The numbers inside the squares represent D′ values as percentages. Red square indicate SNP pairs in strong LD (D′ = 1) with LOD scores below 2, while white squares represent pairs with D′ less than 1 and LOD scores under 2. Analyze more than one polymorphism can either expand the knowledge about the region and also carry information from SNPs in LD.

The pairwise Linkage Disequilibrium (LD) between the *MPO* polymorphisms in the studied groups is shown in Figure 1. We found higher LD between the SNPs rs2071409 and rs2243828 in patients with gestational hypertension (D′ = 0.546, LOD = 2.17, r2 = 0.061) compared to the other groups.

## 3. Discussion

To date, no studies have investigated the impact of *MPO* gene polymorphisms or haplotypes on plasma MPO levels in hypertensive disorders of pregnancy, our main novel findings were that (1) TT genotype for c.–765T>C (rs2243828) polymorphism was associated with lower MPO concentrations than TC+CC genotypes in GH; (2) the CC genotype for the c.–765T>C (rs2243828) polymorphism was found at a higher frequency in the GH group than in the HP group; (3) the ‘C, C’ haplotype may protect against PE and was associated with lower MPO levels in PE, and that (4) the AC+CC genotype for g.9890A>C (rs2071409) polymorphism was associated with lower MPO concentrations in the GH group, when compared to the AA genotype.

Findings on maternal circulating MPO levels in PE have been inconsistent. A study evaluating patients with PE + intrauterine growth restriction showed significantly higher plasma MPO levels before delivery in this group than in healthy pregnant women [18]. Consistent with this finding, other studies have shown that MPO levels are significantly increased in the circulation [24] and placenta of women with PE [22]. On the other hand, in a previous study, our group evaluated 30 cases who developed PE, which included 14 severe cases and 16 mild cases, alongside 57 controls, and reported no significant differences in MPO concentrations in either plasma or urine among the groups [30]. Other studies have also shown no difference in MPO levels [19,20,21] between PE patients and controls. While we cannot offer a definitive explanation for these results, they may be due to variations in the populations studied, differences between plasma and serum samples, variability in the sensitivity and specificity of the immunoassays used [31], or differences in gestational age at the time of sampling.

Although no previous study has examined the effects of *MPO* polymorphisms or haplotypes on circulating MPO levels in HDP, both c.–765T>C (rs2243828) and c.–765T>C (rs2243828) polymorphisms are likely to affect transcription factor binding according to Score 1f at RegulomeDB [28] and they are also classified as candidate cis-Regulatory Elements (cCREs) according to Encyclopedia of DNA Elements (ENCODE) data [29]. In this study, we showed for the first time that in GH patients, the TT genotype for the rs2243828 polymorphism was associated with lower MPO concentrations compared to the TC+CC genotypes (Table 4). At the same time, the ‘C, C’ haplotype was associated with lower MPO levels in PE (Table 5). Together, the AC+CC genotype for g.9890A>C (rs2071409) polymorphism was associated with a lower MPO concentration in the GH group, when compared to the AA genotype (Table 4). Overall, these findings suggest that *MPO* polymorphisms and haplotypes have a significant influence on MPO levels. Although functional studies have not yet confirmed how the c.–765T>C (rs2243828) and g.9890A>C (rs2071409) variants impact *MPO* expression, it is likely that additional factors beyond genotype also play a role in regulating *MPO* expression. Nevertheless, our data indicate that the ‘C, C’ haplotype may offer protection against preeclampsia, potentially due to its association with reduced MPO levels. Neutrophils and their associated proteins, such as MPO, play a key role in regulating proper placental development. Consequently, MPO release, uptake, and function may serve as promising therapeutic targets for managing pregnancy-related complications [11]. However, these reactive compounds can also promote host tissue damage and the development of disease [7,8]. MPO is associated with the impaired bioavailability of NO [10,12], a molecule clinically reduced in PE patients [13,14].

As far as we are aware, no prior research has investigated the association between *MPO* polymorphisms or haplotypes and hypertensive disorders of pregnancy (HDP). Our findings demonstrate for the first time that the CC genotype of the c.–765T>C (rs2243828) polymorphism occurs more frequently in patients with GH compared to healthy pregnant (HP) women (Table 4). Our findings suggest that the CC genotype may be important to GH patients because of its frequency and the fact that the TT genotype was associated with lower MPO levels compared to the TC+CC genotype (Table 4). Increased levels of MPO in the bloodstream are linked with inflammation and heightened oxidative stress [17]. However, the ‘C, C’ haplotype was associated with lower MPO concentration in PE (Table 5). In this context, other *MPO* polymorphisms have also been investigated regarding their effects on MPO levels and associations with diseases. The -463G>A (rs2333227) polymorphism was already associated with susceptibility to developing PE in a Turkish population [27]. The G allele of the same polymorphism was previously investigated as a genetic marker for Kawasaki disease risk in Taiwanese children and was associated with higher MPO levels [32]. The GG genotype was also found to be associated with an increasing amount of serum MPO in chronic lymphocytic leukemia and multiple myeloma [33]. PE shares metabolic risk factors with CVD, such as endothelial dysfunction and inflammation [16], and elevated levels of MPO are linked to poor prognosis and increased risk of CVD-related mortality [34]. Additionally, MPO was proposed as a biomarker for cardiovascular risk prediction [35]. However, in this study, we did not explore the association between MPO levels and other key factors involved in PE, such as endothelial dysfunction and oxidative stress [36,37], and this hypothesis remains to be proved.

Finally, we investigated the LD between the two *MPO* polymorphisms. Here we demonstrated a higher LD between g.9890A>C (rs2071409) and c.–765T>C (rs2243828) polymorphisms in patients with GH (D′ = 0.546, LOD = 2.17, r2 = 0.061) than in the other groups. However, we did not examine other *MPO* polymorphisms, such as the -463G>A (rs2333227) in the promoter region, which may also affect MPO levels. Notably, our results require validation in future studies. These observations are significant, as the identified genetic markers could enhance our understanding of MPO as a potential biomarker for PE.

This study has several limitations. Firstly, the sample size was relatively small, and MPO levels were measured in an even smaller subset of participants. Nonetheless, we observed an association between the c.–765T>C (rs2243828) polymorphism and MPO levels in patients with gestational hypertension, as well as a link between the g.9890A>C (rs2071409) variant and MPO concentrations in the same group. Secondly, we did not assess MPO levels in placental or other tissues, so the extent of its effects at the tissue level remains unclear. Additionally, we did not explore the association between MPO levels and endothelial function, oxidative stress, or antiangiogenic markers. A correlation analysis among these markers could improve understanding of the role of this molecule during PE development and physiopathology, and further studies are needed.

In summary, this is the first study to provide evidence that *MPO* genotypes and haplotypes influence circulating MPO levels in hypertensive disorders of pregnancy and may contribute to disease susceptibility. Our results suggest that *MPO* polymorphisms could serve as potential markers for identifying women at higher risk of developing hypertensive disorders during pregnancy.

## 4. Materials and Methods

### 4.1. Subjects

In this study, we evaluated 136 HP, 118 GH, and 140 PE patients. Participants were recruited from the Department of Gynecology and Obstetrics clinics at Hospital das Clínicas de Ribeirão Preto, University of São Paulo. All participants provided written informed consent, and the research was approved by the Institutional Review Board of the same institution (reference number 4682/2006; approval date: 20 June 2006). Hypertensive disorders of pregnancy were defined following the American College of Obstetricians and Gynecologists (ACOG) guidelines [3]. Gestational hypertension (GH) was defined as elevated blood pressure (systolic ≥ 140 mmHg or diastolic ≥ 90 mmHg) recorded on at least two occasions six hours apart, occurring after 20 weeks of gestation and in the absence of proteinuria (<0.3 g in a 24-h urine sample). Preeclampsia (PE) was characterized by the same criteria for elevated blood pressure (systolic ≥ 140 mmHg or diastolic ≥ 90 mmHg on two readings at least six hours apart) but was accompanied by proteinuria (≥0.3 g/24 h) and/or thrombocytopenia, impaired liver function, pulmonary edema, new-onset headache unresponsive to all forms of management and renal insufficiency with abnormal lab values, all after 20 weeks of gestation [38]. Individuals with hemostatic disorders, chronic hypertension, cancer, multiple pregnancies (twins), or cardiovascular, autoimmune, kidney, or liver diseases were excluded from the study.

### 4.2. Myeloperoxidase Concentration

Plasma MPO levels were quantified using a commercial ELISA kit (Human Myeloperoxidase DuoSet ELISA, R&D Systems, Minneapolis, MN, USA). Samples were diluted at a ratio of 1:100 in reagent diluent (1% BSA in PBS), and the absorbance was measured at 450 nm using a microplate reader (Synergy 4, BioTek, Winooski, VT, USA). The assay’s standard curve ranged from 62.5 to 4000.0 pg/mL.

### 4.3. Genotyping

Genomic DNA was extracted from the cellular component of 1 mL of whole blood using a salting-out method and was stored at −20 °C until analysis. Genotypes for the single nucleotide polymorphisms (SNPs) c.–765T>C in the promoter region (rs2243828; Assay ID: C__16075927_10) and g.9890A>C in the intron 11 (rs2071409; Assay ID: C__15868927_20) of the *MPO* gene were determined using Taqman Allele Discrimination Assays (Applied Biosystems, Carlsbad, CA, USA). Real-time PCR was performed in a 96-well plate with a final volume of 15 µL (2.0 µL of DNA, 6.5 µL of 2X TaqMan Universal PCR Master Mix, 0.75 µL of 20X assay mix). Fluorescence from amplification was detected using a StepOne Plus (Applied Biosystems) device and was analyzed using the manufacturer’s software.

### 4.4. Statistical Analysis

Categorical variables were analyzed using chi-square (χ^2^) tests. For continuous variables, comparisons were made using the Student’s *t*-test or ANOVA, followed by Tukey’s post-hoc test when data were normally distributed, and the Mann–Whitney or Kruskal–Wallis tests, followed by Dunn’s multiple comparisons test, for non-normally distributed data. Correlations were evaluated using either Pearson’s or Spearman’s correlation coefficients, depending on the data distribution. All statistical analyses were conducted using GraphPad Prism version 8.0 for Windows (GraphPad Software, San Diego, CA, USA).

The distribution of genotypes for each polymorphism was assessed for deviation from the Hardy–Weinberg equilibrium, and differences in genotype and allele frequencies were assessed using *χ*^2^ tests. Haplotype frequencies were calculated using the Haplo.stats package version 1.9.7 (available at http://cran.r-project.org/web/packages/haplo.stats/index.html, accessed on 5 February 2025), following previously described methods [39,40]. The possible haplotypes, including the alleles for the two *MPO* SNPs c.–765T>C (rs2243828) and g.9890A>C (rs2071409), were ‘T, A’, ‘T, C’, ‘C, A’, ‘C, C’. Haplotype frequency differences were evaluated using chi-square (χ^2^) tests. Linkage disequilibrium (LD) was determined by calculating D′ values with Haploview software (version 4.2; http://www.broad.mit.edu/mpg/haploview/, accessed on 18 January 2025). *p* < 0.05 was considered statistically significant for all analyses.

When analyzing the effects of *MPO* genotypes on plasmatic levels of myeloperoxidase in HP, GH, and PE groups, we could only measure the rs2243828 SNP in 96 HP, 91 GH, and 99 PE patients, and the rs2071409 SNP in 131 HP, 90 GH, and 96 PE patients. As for the effects of *MPO* haplotypes on plasmatic levels of myeloperoxidase in HP, GH, and PE groups., we could only measure the T,A haplotype in 210 HP, 158 GH, and 210 PE patients; the T,C haplotype in 12 HP, five GH, and 12 PE patients; the C,A haplotype in 31 HP, 47 GH, and 47 PE patients; and the C,C haplotype in 11 HP, nine GH, and five PE patients.

## Figures and Tables

**Table 1:** Clinical and biochemical profiles of healthy pregnant (HP) women and patients with gestational hypertension (GH) and preeclampsia (PE), included in the genotyping analysis.

Parameters	HP (n = 136)	GH (n = 118)	PE (n = 140)
Age (years)	23.0 (20.0–28.0)	27.5 (22.7–32.0) *	26.0 (21.3–32.0) *
Race (white, %)	61.9	71.8	71.3
BMI (Kg/m^2^) before pregnancy	22.7 (20.1–26.3)	29.0 (25.2–33.7) *	27.4 (23.3–32.3) *^,#^
Current smoker (%)	11.5	5.2	11.9
SBP (mmHg)	110.0 (100.0–120.0)	130.0 (120.0–140.0) *	130.0 (122.0–140.0) *
DBP (mmHg)	70.0 (70.0–80.0)	80.0 (70.0–90.0) *	90.0 (80.0–94.0) *^,#^
Antihypertensive drugs at blood collection			
α-Methyldopa(%)	NA	73.5	74.0
Nifedipine(%)	NA	0.9	6.3 ^#^
Hydralazine (%)		1.7	0
GA at blood collection (weeks)	37.0 (36.0–39.0)	37.0 (34.0–39.0)	35.0 (32.0–37.0) *^,#^
RBC count (×10^6^/mL)	4.0 (3.7–4.4)	4.1 (3.8–4.4)	4.2 (3.9–4.4)
Hemoglobin (g/dL)	11.9 (10.9–12.7	12.0 (11.2–12.9)	12.0 (11.1–12.7)
WBC count (×10^3^/mL)	11.9 (8.1–13.1)	9.7 (8.1–11.4)	10.0 (8.4–12.0)
Neutrophils (%)	72.5 (69.1–83.7)	69.4 (63.8–74.0) *	70.1 (66.0–74.0) *
Proteinuria (mg/24 h)	ND	151.6 (109.9–205.4)	409.8 (303.2–920.4) ^#^
Plasma nitrite (nM)	115.8 (72.5–166.5)	73.7 (41.9–127.6) *	80.8 (52.1–124.4) *
Whole blood nitrite (nM)	72.6 (49.5–168.6)	74.9 (31.9–101.2)	49.1 (24.5–83.2) *
GA at delivery (weeks)	40.0 (39.0–41.0)	39.0 (38.0–40.0) *	37.0 (35.0–39.0) *^,#^
Newborn weight (g)	3255.0 (3025.0–3650.0)	3225.0 (2900.0–3525.0)	2825.0 (2040.0–3288.0) *^,#^

Data as median (25th–75th centiles) or percentage. Group comparisons for continuous variables were performed using the Kruskal–Wallis test followed by Dunn’s post-hoc analysis, or the Mann–Whitney test with Dunn’s correction. Categorical variables were analyzed using the chi-square test. * *p* < 0.05 compared to the healthy pregnancy group; ^#^ *p* < 0.05 compared to the gestational hypertension group. Abbreviations: BMI—body mass index; SBP—systolic blood pressure; DBP—diastolic blood pressure; GA—gestational age; RBC—red blood cells; WBC—white blood cells; NA—not applicable; ND—not determined.

**Table 2:** Genotypes and allele frequencies for the *MPO* polymorphisms in healthy pregnant (HP), gestational hypertension (GH), and preeclampsia (PE) groups.

Polymorphism	HP (%)	GH (%)	OR (95% CI)	*p* Value	PE (%)	OR (95% CI)	*p* Value
T–765C (rs2243828)							
TT	0.590	0.544	1.000 (Reference)	–	0.656	1.000 (Reference)	–
TC	0.385	0.351	0.989 (0.572–1.709)	0.969	0.290	0.677 (0.396–1.158)	0.153
CC	0.026	0.105	4.452 (1.200–16.52)	0.016 *	0.053	2.597 (0.643–10.48)	0.167
A allele	0.782	0.719	1.000 (Reference)	–	0.802	1.000 (Reference)	–
G allele	0.218	0.281	1.400 (0.916–2.140)	0.119	0.198	0.888 (0.575–1.372)	0.593
9890A>C (rs2071409)							
AA	0.812	0.886	1.000 (Reference)	–	0.870	1.000 (Reference)	–
AC+CC	0.188	0.114	0.556 (0.265–1.166)	0.116	0.130	0.644 (0.323–1.283)	0.208
T allele	0.902	0.925	1.000 (Reference)	–	0.935	1.000 (Reference)	–
G allele	0.098	0.075	0.739 (0.383–1.424)	0.364	0.065	0.636 (0.331–1.224)	0.172

*MPO* represents myeloperoxidase gene; HP, healthy pregnant; GH, gestational hypertension; PE, preeclampsia; CI, confidence interval; OR, odds ratio. * *p* < 0.05 vs. healthy pregnant group.

**Table 3:** *MPO* haplotypes’ relative frequencies in healthy pregnant (HP), gestational hypertension (GH), and preeclampsia (PE) groups.

Haplotype	HP (n = 117 × 2)	GH (n = 118 × 2)	*p*	OR (95% CI)	PE (n = 131 × 2)	*p*	OR (95% CI)
T, A	0.734	0.696	0.404	1.000 (Reference)	0.750	0.531	1.000 (Reference)
T, C	0.047	0.023	0.178	0.578 (0.202–1.648)	0.051	0.811	1.027 (0.427–2.469)
C, A	0.167	0.231	0.068	1.484 (0.910–2.421)	0.184	0.771	1.113 (0.675–1.838)
C, C	0.050	0.048	0.805	0.985 (0.450–2.159)	0.013	0.025 *	0.246 (0.062–0.971)

*MPO* represents myeloperoxidase gene; OR, odds ratio; CI, confidence intervals. * *p* < 0.05 vs. healthy pregnant group.

**Table 4:** Effects of *MPO* genotypes on plasmatic levels of myeloperoxidase in healthy pregnant (HP), gestational hypertension (GH), and preeclampsia (PE) groups.

Polymorphism	Genotype		HP	GH	PE
			Concentration	Concentration	Concentration
rs2243828		TT	15.0 ± 1.7	6.1 ± 0.5	14.0 ± 1.4
		TC+CC	17.3 ± 2.7	37.4 ± 3.5 ^#^	16.8 ± 2.4
rs2071409		AA	22.6 ± 2.5	19.2 ± 1.9	13.7 ± 1.2
		AC+CC	23.3 ± 4.8	8.1 ± 2.3 *	17.8 ± 3.9

Number of subjects for concentration analysis in rs2243828 polymorphism: healthy pregnant = 96, gestational hypertension = 91, preeclampsia = 99, and in rs2071409 polymorphism: healthy pregnant = 131, gestational hypertension = 90, and preeclampsia = 96. * *p* = 0.02 vs. AA genotype; ^#^ *p* < 0.0001 vs. TT genotype.

**Table 5:** Effects of *MPO* haplotypes on plasmatic levels of myeloperoxidase in healthy pregnant (HP), gestational hypertension (GH), and preeclampsia (PE) groups.

Haplotype	HP	GH	PE
	Concentration	Concentration	Concentration
T,A	5.0 ± 0.36	3.3 ± 0.2	4.0 ± 0.2
T,C	3.5 ± 0.5	5.4 ± 1.5	2.7 ± 0.4
C,A	3.2 ± 0.3	3.6 ± 0.4	3.4 ± 0.3
C,C	2.4 ± 0.3	1.9 ± 0.1	2.0 ± 0.2 ^#^

Number of subjects for concentration analysis in T,A haplotype: healthy pregnant = 210, gestational hypertension = 158, preeclampsia = 210; in T,C haplotype: healthy pregnant = 12, gestational hypertension = 5, preeclampsia = 12; in C,A haplotype: healthy pregnant = 31, gestational hypertension = 47, preeclampsia = 47 and in C,C haplotype: healthy pregnant = 11, gestational hypertension = 9, preeclampsia = 5. Data show Median ± S.E.M; ^#^ *p* = 0.045 vs. T,A haplotype.

## Data Availability

The raw data supporting the conclusions of this article will be made available by the authors on request.
